# Solvent-in-Salt Electrolytes for Fluoride Ion Batteries

**DOI:** 10.1021/acsenergylett.3c00493

**Published:** 2023-05-22

**Authors:** Omar Alshangiti, Giulia Galatolo, Gregory J. Rees, Hua Guo, James A. Quirk, James A. Dawson, Mauro Pasta

**Affiliations:** † Department of Materials, University of Oxford, Oxford OX1 3PH, U.K.; ‡ Chemistry − School of Natural and Environmental Science, Newcastle University, Newcastle upon Tyne NE1 7RU, U.K.

## Abstract

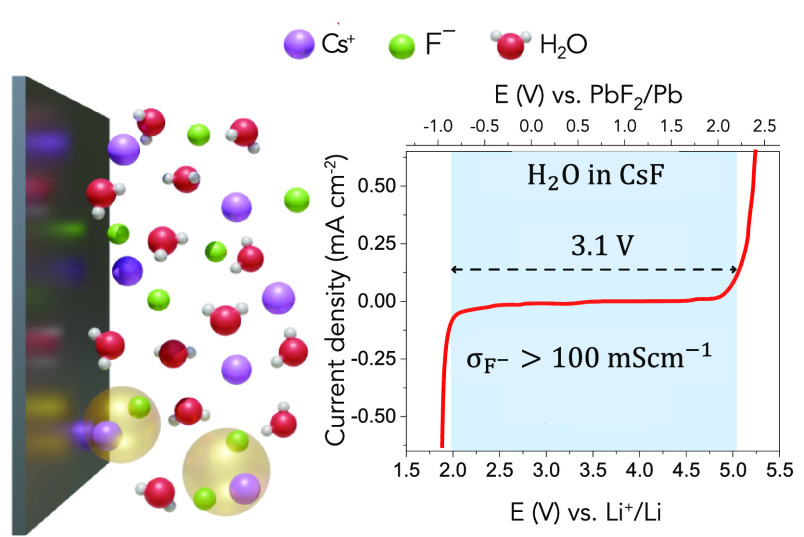

The fluoride ion battery (FIB) is a promising post-lithium ion battery chemistry owing to its high theoretical energy density and the large elemental abundance of its active materials. Nevertheless, its utilization for room-temperature cycling has been impeded by the inability to find sufficiently stable and conductive electrolytes at room temperature. In this work, we report the use of solvent-in-salt electrolytes for FIBs, exploring multiple solvents to show that aqueous cesium fluoride exhibited sufficiently high solubility to achieve an enhanced (electro)chemical stability window (3.1 V) that could enable high operating voltage electrodes, in addition to a suppression of active material dissolution that allows for an improved cycling stability. The solvation structure and transport properties of the electrolyte are also investigated using spectroscopic and computational methods.

The strained supply and unforgiving costs of the critical minerals (Li, Ni, and Co) used in conventional lithium ion batteries (LIBs) have motivated an increasing interest in beyond-lithium battery chemistries.^1^ Fluoride ion batteries (FIBs) utilize the fluoride shuttle between two electrodes with different (de)fluorination potentials.^2^ The large reduction potential for the fluoride ion, attributed to its high electronegativity, promises an electrochemical stability that allows for high operating voltages, whereas the single charge and small ionic radius enable excellent transport properties with minimal polarization compared to multivalent charge carriers (e.g., Mg^2+^, Ca^2+^, Al^3+^), in addition to the economic and environmental advantages allowed by the high fluoride elemental abundance.^3^ Furthermore, the use of conversion electrodes based on transition metal fluorides can provide theoretical energy densities of up to 1393 Wh L^–1^ (588 Wh kg^–1^) enabled by the multielectron conversion and the high theoretical capacity of the transition metals.^4^ These merits, however, have not yet been fully realized for reversible and high energy density FIBs at room temperature (RT), with most of the previously reported FIBs using solid-state electrolytes at operating temperatures above 80 °C,^2,5,6^ and more recently at 60 °C,^7^ despite many efforts to discover and study RT fluoride conductive materials.^8−11^

In contrast, liquid electrolytes are expected to have higher ionic conductivities that enable RT FIBs, in addition to their improved interfacial contact and compatibility with commercial LIBs manufacturing. However, their progress in FIBs has been hindered by two factors, namely, the low solubility of the fluoride salts in the electrochemically stable aprotic organic solvents and the chemical reactivity of the fluoride ion, possibly forming the corrosive hydrofluoric acid (HF) in the presence of any acidic hydrogen.^3,12^ The two most successful liquid electrolyte designs thus far have been based on a synthesized quaternary ammonium fluoride salt dissolved in a partially fluorinated ether^13^ and cesium fluoride (CsF) solubilized in tetraglyme using boron-based anion acceptors.^14−17^ The former exhibited a 4.1 V electrochemical stability window (ESW) and a fluoride solubility of 2.1 M, but suffered from a low ionic conductivity (<3 mS cm^–1^) and significant active material dissolution that resulted in fast capacity fading of the CuF_2_ cathode. On the other hand, electrolytes based on anion acceptors suffered from a similarly low ionic conductivity, a moderate ESW (ca. 2 V), and a low fluoride solubility of less than 0.5 M despite using stoichiometric amounts of the anion acceptor. Other electrolytes based on polymers^18^ or ionic liquids^14^ showed even lower conductivity or electrochemical stability.

Herein, we report solvent-in-salt electrolytes for FIBs, where the low activity of the free solvent molecules results in an enhanced (electro)chemical stability and suppression of the active material dissolution, enabling more stable cycling of higher potential cathodes. At sufficiently high concentrations, the free solvent molecules and the solvent-separated ion pairs present in a traditional salt-in-solvent electrolyte evolve into contact ion pairs and ion aggregates (Scheme 1), resulting in an ion-dominated electrolyte that increases the electrochemical stability at the interface.^19,20^

**Scheme 1:**
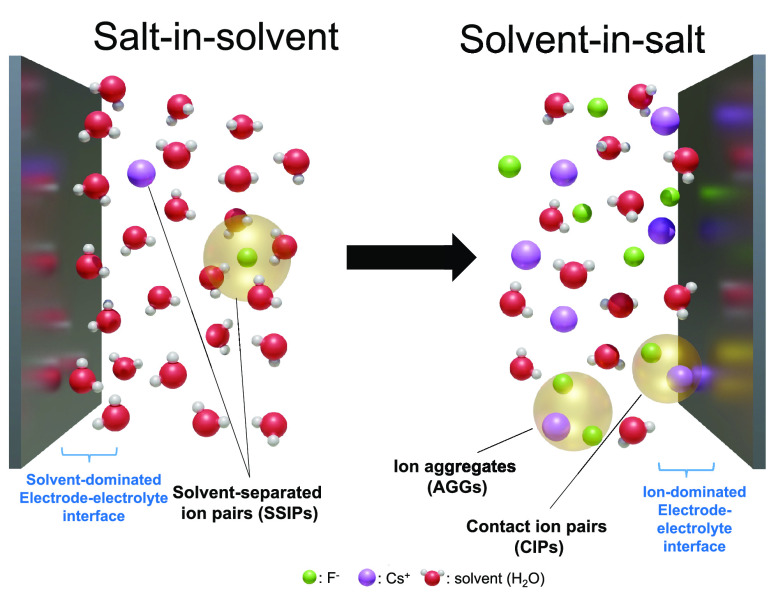
Representation of the Evolution of the Ionic Species in the Bulk and the Electrode–Electrolyte Interface as the Concentration Is Increased from Salt-in-Solvent to Solvent-in-Salt Electrolytes

The solubility of CsF, a cost-effective and readily available fluoride source compared to organic-based salts, was first measured at RT in a range of protic solvents using inductively coupled plasma mass spectrometry (ICP-MS). Water was found to have by far the highest solubility of around 37 molal (*m*) (Figure 1a). Calculating the salt-to-solvent weight and volume ratios as a function of concentration showed that water was the only solvent that satisfied the theoretical solvent-in-salt condition defined by Suo et al.^21^ where both ratios exceed unity (Figure 1c). This also showed that this condition could not be satisfied for less soluble salts, such as potassium fluoride (KF), where the salt-to-solvent ratio would only exceed unity beyond the solubility limit. The high solubility of CsF in water resulted in a clear liquid electrolyte with a solvent-to-salt molar ratio of 1.7 for the saturated solution, resembling a “hydrate melt” at RT. This choice of solvent over other protic solvents was further aided by the advantageous properties of water such as the higher boiling point, inflammability, and nontoxicity, in addition to eliminating the need for ultradry cell manufacturing conditions.

**Figure 1:**
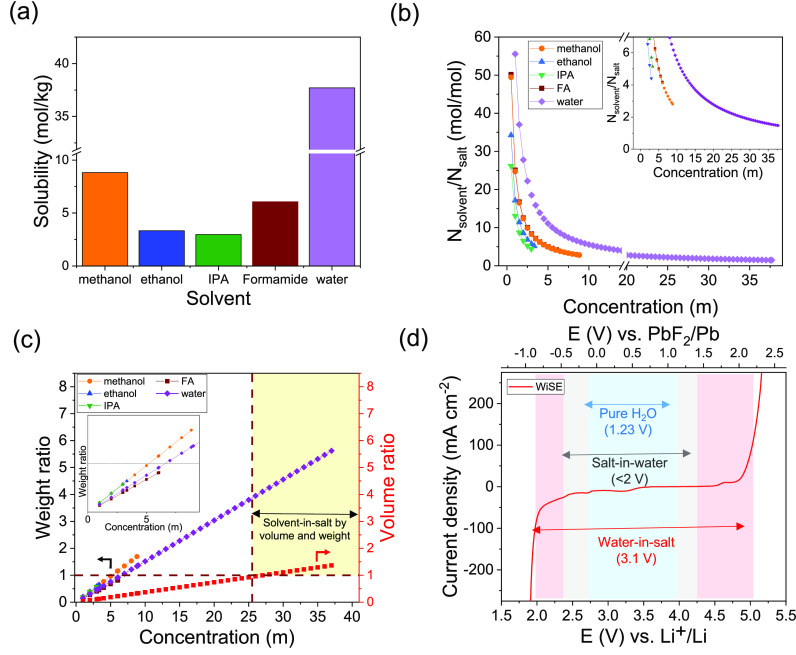
Solubility of CsF determined using ICP-MS in common protic solvents (a), solvent-to-salt molar ratio showing <2 coordination number for CsF in water (b), salt-to-solvent weight and volume ratios showing the concentration range of “solvent-in-salt” (c), and electrochemical stability window expansion in the water-in-salt electrolyte (this work) compared to pure water and other aqueous electrolytes (d).

Aqueous electrolytes, however, are known to have a narrow ESW, inhibiting the use of high operating voltage electrode pairs and limiting the energy density. To explore the effect of water-in-salt electrolytes (WiSEs) on expanding the ESW, linear sweep voltammetry was performed on multiple concentrations. As the concentration was increased beyond 25 *m*, the stability window was shown to expand to around 3.1 V (Figure 1d). This enhancement in the ESW is a result of the decrease in the activity of free water molecules and the resulting change in the solvation structure, where the electrode–electrolyte interface becomes dominated by ionic species instead of the electrochemically unstable free water molecules. Very recently, a concentrated aqueous electrolyte was reported for a FIB but exhibited limited expansion in the ESW (2.1 V),^22^ likely due to the presence of a large fraction of free water molecules imposed by the lower solubility of KF (Figure S1).

Since solvent-in-salt electrolytes are expected to possess high viscosities that hinder ionic transport, the ionic conductivity and diffusivity were measured using electrochemical impedance spectroscopy and pulsed field gradient nuclear magnetic resonance (PFG NMR) spectroscopy, respectively. The ionic conductivity was found to peak at around 10 *m*, with the 25 *m* electrolyte exhibiting a conductivity of 152 mS cm^–1^ (Figure 2a), 2 orders of magnitude higher than for previously reported FIB organic-based electrolytes.^4^ The fluoride and cesium ion diffusivities dropped from 2.45 × 10^–9^ and 2.70 × 10^–9^ m^2^ s^–1^, respectively, for the 1 *m* electrolyte to 2.51 × 10^–10^ and 1.24 × 10^–10^ m^2^ s^–1^, respectively, for the 25 *m* electrolyte due to the increased viscosity (Figure 2b). However, the relative fluoride ion diffusion was shown to significantly improve at higher concentrations as evident by the higher transport number (Figure 2b). This improved fluoride mobility is likely attributed to a stronger O–Cs complexation in the concentrated electrolyte as the competing O–H solvent–solvent interaction is weakened due to the lower water activity. ^17^O NMR corroborated this postulate by showing the water oxygen peak at higher chemical shifts, indicating a more deshielded oxygen, in addition to a pronounced broadening indicating “solid-like” and less mobile water molecules (Figure 3a), whereas ^1^H NMR showed the reverse trend (Figure S2), indicating a weakening solvent–solvent O–H bond and a strengthening O–Cs bond. This nearly immobile water network strongly complexing the cesium ion allows for a freer fluoride ion migration. Similar improved anionic migration was previously suggested in studies of lithium ion electrolytes, where the cation is known to bind more strongly to the solvent compared to the anion.^23^

**Figure 2:**
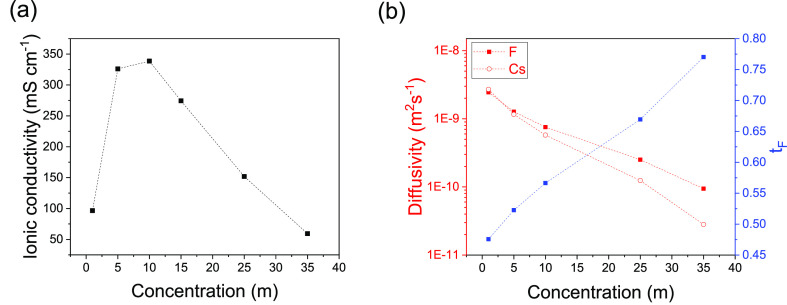
Ionic conductivity (a) and diffusion coefficient for the fluoride and cesium ions and the fluoride transport number (b) as a function of concentration.

**Figure 3:**
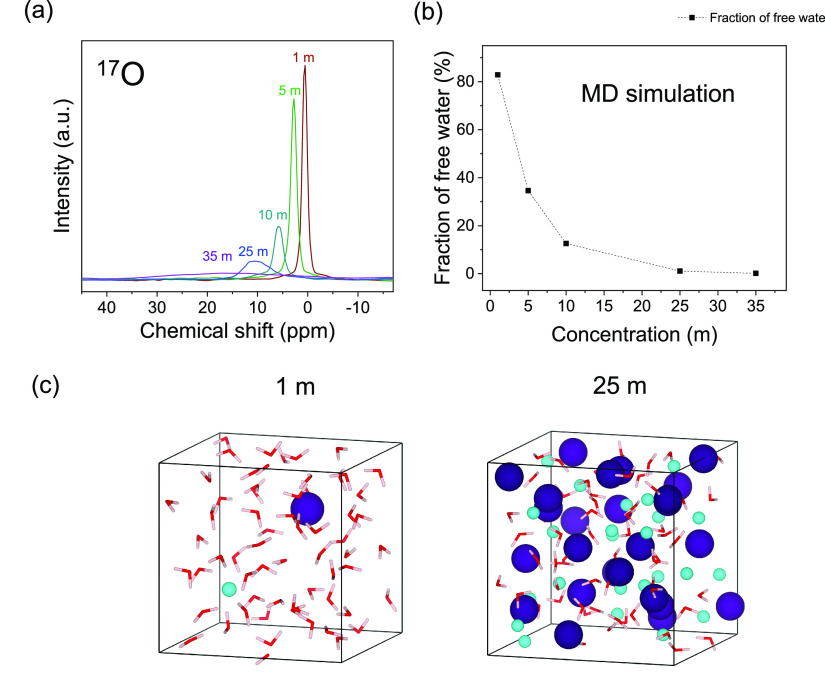
Solvation properties for the water-in-salt electrolyte. ^17^O NMR spectroscopy showing the peak broadening and peak shift as the water molecules become less mobile (a), fraction of free water molecules calculated from the MD simulation (b), and MD relaxed structure at 1 and 25 *m* showing the formation of contact ion pairs and aggregates and elimination of free water molecules (c).

The solvation structure was characterized using spectroscopic and computational methods to explore the evolution from solvent–ion to ion–ion dominated interactions (Figure 3). The molecular dynamics (MD) simulation showed an increased presence of ion pairs and aggregates in the concentrated electrolyte (Figure 3c, Figure S3), in addition to a rapid decrease in the fraction of free water molecules (Figure 3b). This change in the solvation structure was reflected in the ^17^O NMR spectra for the water molecule, where the increased deshielding was due to a higher fraction of the water molecules donating their oxygen electrons to the Cs ions, and supported the expansion in the ESW (Figure 1d).

The chemical stability of the fluoride ion has been a major concern in FIBs, where incautious electrolyte design can result in the fluoride ion forming corrosive HF. In protic media, the fast hydrogen exchange due to the HF/F^–^ equilibrium is known to result in a single ^19^F NMR signal with a chemical shift at the average position of all the fluoride species,^24^ making ^19^F NMR inadequate in quantifying the HF content in protic solvents. Diluted aqueous fluoride electrolytes are, however, still expected to have some equilibrating HF given the p*K*_a_ value of 3.8, despite this not preventing their stable and reversible cycling for over 1000 cycles in previous reports.^25^ The HF fraction was therefore calculated from the proton activity measurement given the validity of potentiometric pH measurements at ultrahigh concentrations (>17 *m*).^26^ The HF content was found to decrease to near zero in the WiSEs as the water molecules donating the protons became more scarce and the solution more basic (Figure 4a, Table S1). This observation is further confirmed by the increasingly deshielded fluoride in the ^19^F NMR (Figure 4b). Despite the difficulty of comparing the “nakedness” of the fluoride ion in multiple solvents due to other factors affecting the chemical shift (solvent electric dipole, magnetic anisotropy, etc.), comparing chemical shifts across the same solvent is a valid proxy for the fluoride solvation environment.^27^ In this case, as the concentration is increased, the fluoride shifts away from the HF region (−160 to −170 ppm),^24^ increasingly resembling the naked, less coordinated fluoride in organic solvents. Furthermore, this chemical stability was accompanied by an increase in thermal stability, with a higher decomposition temperature onset and wider liquid range due to the suppressed melting point, for the WiSE (Figure S4).

**Figure 4:**
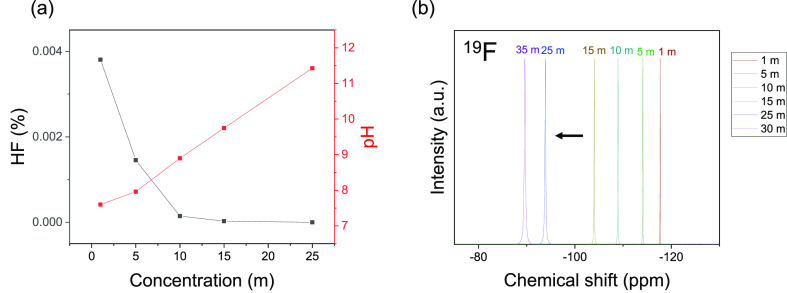
Fluoride ion chemical species and electronic environment. pH as a function of concentration indicating the decay of the HF content (a) and ^19^F NMR spectroscopy (b).

Finally, to explore the RT cycling performance for the WiSE, galvanostatic cycling and CV were performed for the 25 *m* electrolyte, given that it exhibited an excellent trade-off between the water-in-salt properties (wide ESW and low water fraction) and transport properties (diffusion coefficient and ionic conductivity). Symmetric Pb|PbF_2_ coin cells were cycled at a C/10 rate, with the low-concentration electrolyte showing faster capacity fading (Figure 5a). This is attributed to the aggravated active material dissolution at lower concentrations (given the constant solubility product, *K*_sp_, lower fluoride concentrations will result in higher metal ion dissolution), a problem known to be detrimental in previous FIBs based on conversion of metal fluorides.^13,28,29^ The WiSE, however, showed improved and more stable capacity retention, with this differentiated cycling performance expected to become more evident with further cycling.^30^ For this symmetric cell, increased cycling showed a complete capacity loss for the diluted electrolyte by the 100th cycle (Figure S5). The cycling of the WiSE was further demonstrated for CuF_2_, where CV showed 30 cycles, compared to previous CV reports of the CuF_2_ where the dissolution resulted in full capacity loss by the 10th CV cycle.^13^ The significantly suppressed CuF_2_ dissolution was also visually observed as the blue color attributed to the dissolved [Cu(H_2_O)]^2+^ being eliminated at the 25 *m* electrolyte (Figure S6). CuF_2_ is considered to be the holy grail of cathode materials in FIBs due to its high reduction potential and high capacity (528 mAh g^–1^),^3,13^ and a WiSE can be the system to allow for its RT stable cycling. Furthermore, the cycling of AgF_2_ was shown in a fluoride shuttle system with a potential exceeding 4 V (vs Li^+^/Li), along with the cycling of ZnF_2_ closer to cathodic limit of the ESW (Figure 5c). With further optimization of particle size, cathode fabrication, and careful selection of the anode materials, WiSEs have the merits to allow for further study of the fluorination mechanisms and allow for high-voltage (>2 V) FIB cycling.

**Figure 5:**
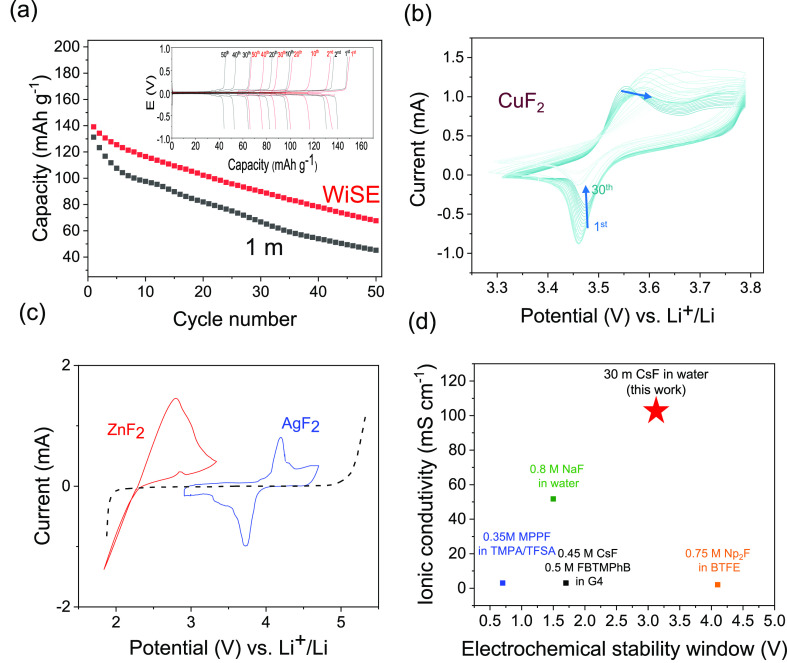
Galvanostatic cycling of symmetric Pb|PbF_2_ at 1 and 25 *m* showing improved capacity retention in WiSE (a). Cyclic voltammetry of CuF_2_ in the 25 *m* electrolyte (b) and of AgF_2_ and ZnF_2_ near the oxidative and reductive limits of WiSE (c). Performance comparison with selected fluoride ion battery electrolytes based on ESW and conductivity (d).

In conclusion, the water-in-salt electrolyte was shown to exhibit room-temperature transport properties and (electro)chemical stability that has been lacking in most fluoride ion battery electrolytes. NMR spectroscopy for the fluoride ion coupled with pH measurements showed a nearly complete suppression of hydrogen fluoride formation and thus an increased chemical stability. MD simulations and ^17^O NMR shed light on the solvation structure and showed the elimination of free solvent molecules, confirming the mechanism behind the expanded electrochemical stability window. Finally, our preliminary study of the cycling performance showed an increased capacity retention for the concentrated electrolyte, allowing for a more stable cycling and suppression of active material dissolution and offering a path for the cycling and study of the fluorination conversion mechanism for high-capacity cathode materials such as CuF_2_.

## Supporting Information Available

Materials and experimental methods for the electrochemical testing, NMR spectra, and computational calculations; supplemental figures including ^1^H NMR spectra, LSV of salt-in-solvent electrolyte, Radial distribution function of MD claclulation, DSC and TGA measurements, and visual characterization of the CuF_2_ dissolution (PDF)
