# Supplementary material for: Solvent-in-Salt Electrolytes for Fluoride Ion Batteries
Source: ACS Energy Lett. 2023 May 22;8(6):2668–73. doi: 10.1021/acsenergylett.3c00493 (PMC10262201; doi:10.1021/acsenergylett.3c00493)
Supplement: Supplementary file 1 — nz3c00493_si_001.pdf [file nz3c00493_si_001.pdf]

Solvent-in-salt electrolytes for fluoride ion batteries

Omar Alshangiti,† Giulia Galatolo,† Gregory J. Rees,† Hua Guo,† James A. Quirk,‡ James A. Dawson,‡ and Mauro Pasta*,†

†*Department of Materials, University of Oxford, OX1 3PH, UK*

‡*Chemistry – School of Natural and Environmental Science, Newcastle University, Newcastle upon Tyne NE1 7RU, UK*

E-mail: mauro.pasta@materials.ox.ac.uk

Phone: +44 01865273777

Supporting information

Experimental

A. Materials

Cesium fluoride (CsF) was purchased from sigma and used without further processing. Lead was synthesized from lead acetate (sigma) by thermal decomposition at 500 °C under argon for 25 minutes to obtain fine powdered Pb. Copper fluoride (thermofischer scientific) and lead fluoride (sigma) were ball milled for 12 and 15 hours, respectively, at 700 rpm and 3/7 min work/rest cycles. PTFE powder for the active material binder was purchased from Goodfellow Cambridge (675 um particle size) and used without further processing. Cell components for the 1032 coins were purchased from pi-kem.

B. Linear sweep and cyclic voltammetry:

LSV and CV were performed on the corresponding concentration of electrolyte using a scan rate of 1 mV/s in a 3-electrode cell made from Teflon. The LSV for the electrochemical stability window was performed using a glassy carbon working electrode, fluorinated lead pseudo-reference electrode (2.84 vs. Li^+/Li, calibrated using ferrocene), and a silver wire counter. The CV of the active material was performed with the active material casting as the working electrode. The cycling was performed and recorded using VMP3 Biologic potentiostats.

C. ICP-MS solubility measurements:

An oversaturated CsF solution in the corresponding solvent was stirred at ambient temperature (recorded 23 °C) overnight and then filtered using a syringe filter. The filtrate was diluted and digested in diluted nitric acid solution. The Cs content was measured using ICP-MS and the absolute concentration determined using a Cs standard calibration curve.

D. Nuclear Magnetic Resonance (NMR):

^{17}O NMR spectra was obtained using direct observation of natural abundance ^{17}O at 11.75 T ($\nu_0(^{17}O) = 67.77$ MHz) on Bruker Avance III spectrometer equipped with a 5 mm smart probe. All spectra were referenced to $H_2{}^{17}O$ ($\delta_{iso} = 0$ ppm), with a relaxation time of 200 ms, and 1024 transients collected per experiment. Diffusion coefficients were measured at 30 °C using pulsed field gradient (PFG) NMR, with measurements completed at 9.45 T (ν_0(1H) = 400.19 MHz, $\nu_0(^{19}F) = 376.51$, and $\nu_0(^{133}Cs) = 52.49$ MHz) on Bruker Avance III HD spectrometer using a 5 mm single-axis diffusion probe with exchangeable ceramic heads. A stimulated echo pulse sequence was utilized for all PFG experiments with effective gradient pulse durations (δ) ranging between 1 to 1.5 ms, and a diffusion time (Δ) of 10-30 ms, with the gradient amplitude varying between 0.1 and 24 T/m in 36 steps. All samples were sealed

in a J-Young air-tight NMR tube, temperature stabilized to 303.1 K, and a 1-second recycle delay was used throughout. All data was fitted to the Stejskal-Tanner equation:

$$f(x) = I_0 e^{-\gamma^2 g^2 \delta^2 \frac{(\Delta-\delta)}{3} D} \tag{1}$$

An R^2 value of 0.999, errors of $< 1\%$ and normal distribution of the residuals (>0.5 Shapiro Wilko score) were observed for all experiments.

E. Ionic conductivity measurement:

Electrochemical impedance spectroscopy was performed using a homemade PEEK cell fitted with two stainless steel blocking electrodes. The cell was equilibrated at 30 °C for 30 min before the frequency was varied from 1 mHz to 1 MHz, with the ionic conductivity calculated from the linear fit of the Nyquist plot.

F. Galvanostatic cycling:

1032-coin cells were cycled using Biologic VMP3 potentiostats. The casting was prepared by dry-mixing of the corresponding ratios of active material, conductive carbon fiber, and PTFE powder. For the $PbPbF_2$ cell, this corresponded to 48 %wt Pb, 48%wt PbF_2, 3%wt carbon fiber, and 1%wt PTFE, rolled into 40 μm thick electrodes. The coin cells were (dis)charged at a rate of C/10 (current density of 12.9 μA mg^{-1}) and cutoff potentials of $\pm$ 0.8 V. During cell assembly, an electrolyte volume of ca. 200 μL was used.

G. Computational parameters:

All calculations were carried out using VASP. Molecular dynamics (MD) was accelerated through the use of on-the-fly trained machine-learned forcefields (MLFF) as implemented in VASP6.[1] The cutoffs for the radial and angular descriptors were both 5 Å. These parameters are sufficient to yield root-mean-squared errors in the force of less than 0.1 eV/Å and in the

energy of less than 1 meV. A comparison of radial distribution functions produced from pure ab-initio MD and from MLFF MD shows that the MLFF is accurate enough to produce near-indistinguishable interatomic distances (Figure S3) 3.

For ab-initio calculations, we used the semilocal RPBE exchange-correlation functional with D3 dispersion corrections.[2,3] Projector-augmented wave pseudopotentials[4] are utilised with $1s^1$, $2s^2 2p^4$, $2s^2 2p^5$, and $5s^2 5p^6 6s^1$, as valences for H, O, F, and Cs, respectively. A plane-wave cutoff energy of 600 eV is employed with Γ-point only sampling in reciprocal space.

Initial geometries were generated with PACKMOL,[5] with 58 water molecules in each system and an appropriate number of Cs and F ions for each considered concentration. Each initial geometry was equilibrated at 500 K for 5 ps in an NVT ensemble, then at 300 K for 5 ps in an NPT ensemble, with production simulations running for at least 25 ps in an NPT ensemble. Water molecules within a radial cutoff of either 3.6 Å for Cs or 2.9 Å for F were determined to be bound, hence the fraction of free water could be determined.

For reproducibility, we provide an example VASP input file (INCAR) for on-the-fly training of the MLFF:

```
#Basic parameters
GGA = RP
IVDW=11
ALGO = A
ENCUT = 600
ISMEAR = 0
SIGMA = 0.05
LREAL = Auto
ISYM = 0
NELM = 300
EDIFF = 1E-6
```

```
AMIX = 0.05

#Parallelization of ab initio calculations
NCORE = 16
KPAR = 1

#MD
IBRION = 0
MDALGO = 3 # NPT
ISIF = 3
LANGEVIN_GAMMA = 4*10.0
LANGEVIN_GAMMA_L = 1. 1. 1.
SMASS = 1.0
TEBEG = 300
TEEND = 300
NSW = 100000000
POTIM = 1.0

#Machine learning paramters
ML_LMLFF = .TRUE.
ML_ISTART = 1 # On the fly training
ML_RCUT1 = 5.0
ML_RCUT2 = 5.0
ML_MB = 2500

# Cs and F are more slowly sampled
# Adding this flag with discard old O and H
```

```
# configurations
ML_LBASIS_DISCARD=.TRUE.
```

H. pH measurement and HF-content calculation:

The pH measurements were performed using an ETI 8100 plus pH meter calibrated using pH 7 and 10 standard solutions. The HF fraction in the electrolyte was calculated as follows:

$$K_a = \frac{a_{\mathrm{F^-}} a_{\mathrm{H^+}}}{a_{\mathrm{HF}} a_{\mathrm{H_2O}}} \tag{2}$$

with rearranging:

$$\frac{a_{\mathrm{HF}}}{a_{\mathrm{F^-}}} = \frac{K_a a_{\mathrm{H_2O}}}{a_{\mathrm{H^+}}} \tag{3}$$

$$f_{HF} = \frac{a_{\mathrm{HF}}}{a_{\mathrm{F^-}} + a_{\mathrm{HF}}} = \frac{1}{\frac{K_a a_{\mathrm{H_2O}}}{a_{\mathrm{H^+}}} + 1} = \frac{a_{\mathrm{H^+}}}{K_a a_{\mathrm{H_2O}} + a_{\mathrm{H^+}}} \tag{4}$$

The H^+ activity was measured directly from the potentiometer. The water activity was estimated from the concentration and the salt density:

Table S1: Calculated water concentration at the corresponding CsF concentrations.

CsF concentration (mol/kg)	$[H_2O]$ (mol/L)[a]
1	53.58
5	46.91
10	40.59
15	35.77
25	28.91

[a] In this case, the water activity is only an estimate due to the inevitable discrepancy between $[H_2O]$ and $a_{\mathrm{H_2O}}$. However, the fast decay of %HF would still hold given the exponential decay of the measured $a_{\mathrm{H^+}}$(Figure 3a) compared to the linear decay of the water concentration.

Supplementary Figures

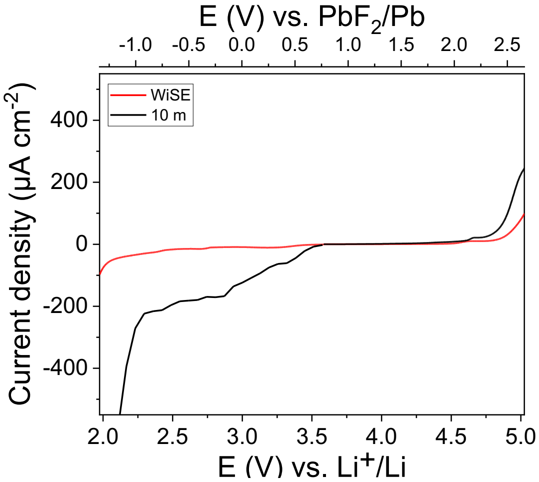

Figure S1: Linear sweep voltammogram for the 10 m electrolyte showing less passivation and electrochemical stability, with an ESW of 2.06 V (based on a 150 μA cm^{-2} cutoff).

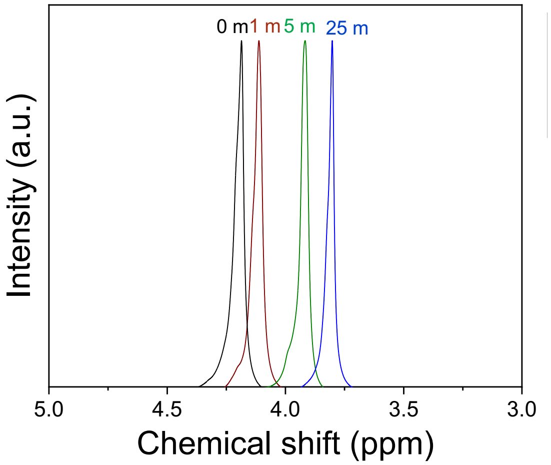

Figure S2: ^{1}H NMR spectra for 0, 1, 5, and 25 m showing the hydrogen peak shifting to higher frequency, opposing the trend observed in the ^{19}F and ^{17}O spectra.

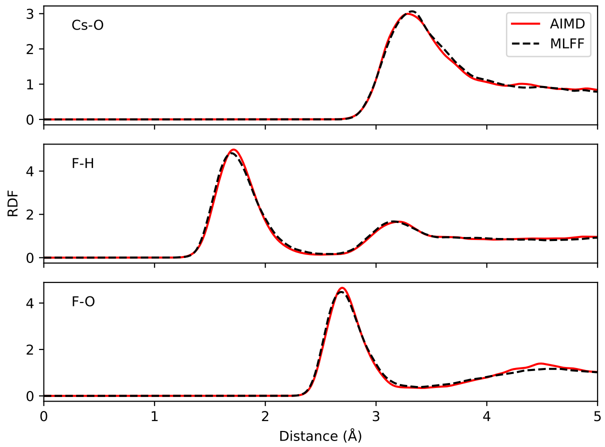

Figure S3: Radial distribution function (using 10 m as a model electrolyte) showing the bond lengths and ion aggregation for the MLFF in agreement with the full ab-initio calculation.

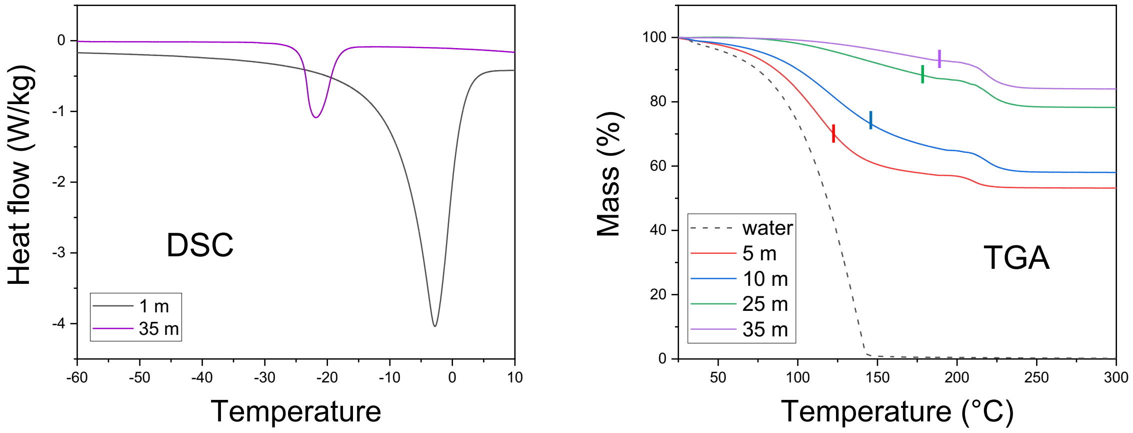

Figure S 4: DSC scans at sub-zero temperatures (a), and TGA measurement at elevated temperature (b), both showing an extended liquid range due to the suppressed freezing point and increased inflection temperature.

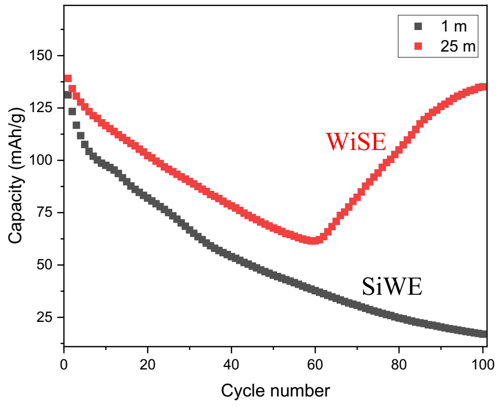

Figure S5: Galvanostatic cycling of symmetric Pb/PbF_2 showing capacity fading of the diluted electrolyte and self-improving capacity of the WiSE, a behaviour which been previously observed and attributed to expansion in the active material network.[6]

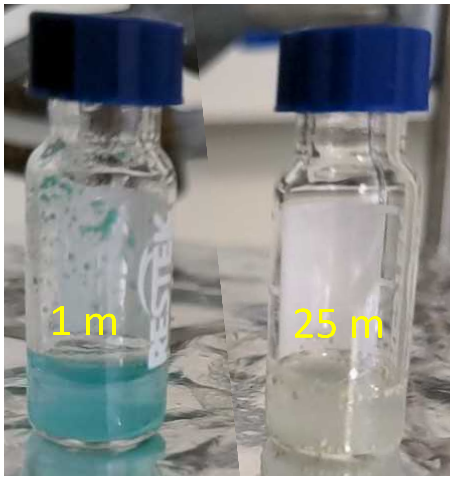

Figure S6: CuF_2 suspension in the 1 and 25 m electrolytes showing the elimination of the blue color (due to the dissolved $[Cu(H_2O)_6]^{2+}$) in the 25 m due to suppressed dissolution in the water-in-salt electrolyte.

References

(1) Jinnouchi, R.; Karsai, F.; Kresse, G. On-the-fly machine learning force field generation: Application to melting points. *Phys. Rev. B* **2019**, *100*, 014105, Publisher: American Physical Society.

(2) Hammer, B.; Hansen, L. B.; NÃžrskov, J. K. Improved adsorption energetics within density-functional theory using revised Perdew-Burke-Ernzerhof functionals. *Phys. Rev. B* **1999**, *59*, 7413–7421, Publisher: American Physical Society.

(3) Grimme, S.; Antony, J.; Ehrlich, S.; Krieg, H. A consistent and accurate ab initio parametrization of density functional dispersion correction (DFT-D) for the 94 elements H-Pu. *J. Chem. Phys.* **2010**, *132*, 154104, Publisher: American Institute of Physics.

(4) Kresse, G.; Joubert, D. From ultrasoft pseudopotentials to the projector augmented-wave method. *Phys. Rev. B* **1999**, *59*, 1758–1775, Publisher: American Physical Society.

(5) Martínez, L.; Andrade, R.; Birgin, E. G.; Martínez, J. M. PACKMOL: A package for building initial configurations for molecular dynamics simulations. *Journal of computational chemistry* **2009**, *30.13*, 2157–2164.

(6) Liu, Y.; Yan, X.; Yu, Y.; Yang, X. Self-improving anodes for lithium-ion batteries: Continuous interlamellar spacing expansion induced capacity increase in polydopamine-derived nitrogen-doped carbon tubes during cycling. *Journal of Materials Chemistry A* **2015**, *3*, 20880–20885.
